# Age and Language Effects on Temporal Cognition in Chinese and English

**DOI:** 10.1002/pchj.70069

**Published:** 2025-12-10

**Authors:** Rong Bao

**Affiliations:** ^1^ Department of Chinese Language and Literature, Faculty of Arts and Humanities University of Macau Macao SAR China

**Keywords:** D‐time, ego face towards, S‐time, temporal focus

## Abstract

Younger and older L1 Chinese speakers differ in where they place their focus—young adults look more to the future, while older adults value the past—yet neither group faces toward the past. Instead, all L1 Chinese participants consistently adopt a future‐facing perspective. When interpreting ambiguous temporal expressions, they rely on S‐Time: “前” (“qian”, front) refers to earlier (past) moments and “后” (“hou”, back) to later (future) moments. This reflects a reference frame of S‐Time rather than a backward orientation toward the past. In contrast, L1 English speakers prefer D‐Time, mapping “front” onto the future and “back” onto the past. Together, these findings show that although age shifts temporal focus among L1 Chinese speakers, cultural and values background determines the dominant reference frames of temporal representations and cognition—S‐Time for L1 Chinese speakers and D‐Time for L1 English speakers.

## 1 Introduction

The temporal metaphor serves as a crucial mechanism for human temporal representation and cognition, exhibiting universality across diverse linguistic systems. However, C. Sinha and V. Sinha's research challenges the universality of the spatialization paradigm for time, demonstrating significant exceptions. C. Sinha et al. (2011) show the Amondawa language lacks spatial mapping for temporal relations and a numerical calendar, proposing the Mediated Mapping Hypothesis. V. Sinha (2019) finds that three indigenous Brazilian languages possess rich event‐based time interval systems but lack metric time units and metaphoric space–time mapping, showing no conceptual timelines or spatialized “past/future.” V. Sinha (2022) emphasizes the limitation of WEIRD‐based research, contrasting metric time (calendar/clock time) with event‐based time organized via lexicalized indices, which may dominate in non‐WEIRD cultures where metric time is absent or subordinate.

These findings, while revealing the limitations of the spatialization paradigm of temporal metaphor, nevertheless underscore that the conceptual mapping from spatial domains to temporal domains generally demonstrates cross‐linguistic and cross‐cultural generality; significant variations exist in temporal directionality representations (Alverson 1994; K. E. Moore 2011; K. E. Moore 2014; Núñez and Sweetser 2006; de la Fuente et al. 2014; Li and Cao 2018; Chen 2021b). This phenomenon has prompted sustained academic discourse regarding the ego towards the past or the future in spatial temporal orientation.

It is widely agreed among native English speakers that the self is oriented toward the future, with the front of the ego representing the future and the back representing the past (Clark 1973; Lakoff and Johnson 1980). This model is based on the theory of embodied cognition, which emphasizes that human physical movement (forward bodily displacement) and visual perception (the dominance of the frontal field) play key roles in shaping our conceptualization of time. As an individual moves along a path, the path already traveled lies behind, while the path yet to be traveled extends ahead—this movement‐based temporal representation forms the cognitive foundation of English spatio‐temporal metaphors (Lakoff and Johnson 1980).

In contrast, the Chinese spatio‐temporal metaphor system exhibits unique complexities. In Chinese, the term “前” (“qian”) can refer either to the past (as in “前年” meaning the year before last) or to the future as in “前途” (“qian tu” meaning prospects), and it tends to denote an earlier time (Chen 2021a; Yang et al. 2023; Chen and Zhang 2021; Bao 2022; Yang and Yang 2024). This contradictory phenomenon has led to controversy among L1 Chinese speakers: in Chinese temporal representation and orientation, is the ego faced and oriented toward the past or the future?

To explain whether the ego in temporal representation tends to orient toward the past or the future, de la Fuente et al. (2014) proposed the Temporal Focus Hypothesis, positing that the degree to which an individual or culture focuses on the past or the future determines the metaphorical spatial mapping of time. If a culture is more future‐focus, then the future is mapped to the “front”; if more past‐focus, then the past is mapped to the “front”. Experiments have shown that Spaniards tend to place the future in front and are more likely to endorse future‐focus statements, whereas Moroccans tend to place the past in front and are more likely to endorse past‐focus statements. This finding was corroborated by cross‐cultural experiments conducted by Li and Cao (2018) between the Han and Qiang in China, which verified how an individual's or culture's focus on the past or future (i.e., temporal focus) influences the implicit direction of spatio‐temporal metaphors (such as “the past in front of the ego” or “the future in front of the ego”). The Temporal Focus Scale is currently one of the most reliable and valid tools for measuring temporal focus, having achieved statistical standards for reliability and validity in countries such as the United States, the United Kingdom, China, and Japan (Shipp et al. 2009; McKay et al. 2012; Chishima et al. 2017; Zhu et al. 2022).

The research described above posits that in languages and cultures with a future temporal focus, the future is placed in front of the ego, whereas in those with a past temporal focus, the past is placed in front of the ego. Beyond cultural differences, it is also worth considering whether age modulates temporal focus within culturally homogeneous groups,. de la Fuente et al. (2014) found that older Spaniards tend to focus more on the past than younger people, and prefer to choose the past in front of them. Bylund et al. (2019) tested young and old Afrikaners and found that the older group had a significantly higher tendency to place the past in front, supporting age as a key factor in spatio‐temporal mapping. These findings prompt further questions: Does age influence the spatio‐temporal mapping of time among native Chinese speakers, thereby affecting their temporal direction and orientation? And within the same culture and language, can different temporal focus lead to variations in the mapping of spatio‐temporal relations? Specifically, does a past‐oriented focus inherently result in the ego facing the past, with the past mapped in front? To address these gaps, we adopt a cognitive‐linguistic approach and examine how age, language, and temporal focus influence Chinese and English speakers' temporal expressions.

### 1.1 Theoretical Frame: S‐Time and D‐Time

**FIGURE 1:**
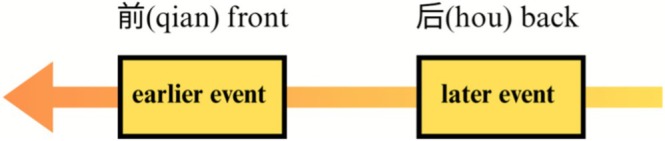
S‐Time.

Within the human cognitive system, time is conceptualized as a sequential continuum in which events are ordered by relative anteriority and posteriority. In this model, events nearer the start of the sequence are construed as earlier, while those nearer the end are construed as later. This constitutes a sequence reference frame for temporal cognition. Many scholars have recognized this frame, referring to it with terms such as sequence position (K. Moore 2006), temporal reference‐point model (Núñez and Sweetser 2006), time‐reference‐point (Time‐RP) (Yu 2012), sequence‐based reference frame (Chen 2021b; Bao 2025), and S‐Time (Le Guen and Balam 2012; Yang et al. 2023). Following Yang et al. (2023), we adopt the label S‐Time to denote this mode of temporal reference. As shown in Figure 1, S‐Time encodes ordinal relations within a sequence (earlier versus later) and operates independently of a privileged “Now” or other deictic center.

**FIGURE 2:**
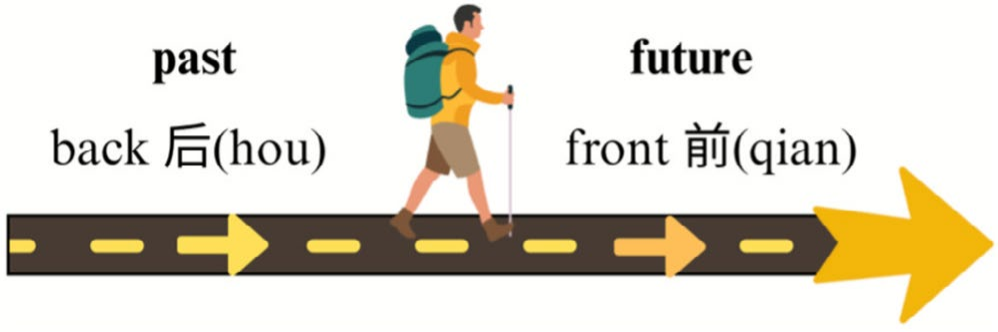
D‐Time.

Ego's perspective can also be projected onto temporal representation, producing an ego reference frame of temporal cognition. Previous research has identified this ego‐based metaphor, labeling it variously as ego‐based metaphor (K. Moore 2006), ego‐reference‐point model (Núñez and Sweetser 2006), ego‐reference‐point (Ego‐RP) (Yu 2012), and D‐Time (Yang et al. 2023). We use D‐Time (Yang et al. 2023) to describe this observer‐centered temporal cognition mechanism. D‐Time is deictic and speaker‐anchored: the interpretation of temporal expressions depends on a dynamic “Now” (i.e., the speaker's or observer's present), thereby generating past–future distinctions and motivating passage metaphors such as MOVING‐EGO and MOVING‐TIME. Under D‐Time, temporal intervals are classified as past, present, or future relative to the ego's immediate vantage, and spatial terms like front and back map onto futurity or pastness according to the ego's embodied orientation (see Figure 2).

### 1.2 Research Questions

Guided by the S‐Time and D‐Time theory introduced above, this study aims to investigate how temporal reference frames are influenced by age, temporal focus, ego orientation, L1 background. We formulate four targeted research questions.

(Experiment 1) Do younger and older L1 Chinese speakers differ in temporal focus (i.e., the tendency to attend to or value the future versus the past)?

(Experiment 2) When adopting D‐Time, do past‐focused and future‐focused native Chinese speakers differ in ego orientation (i.e., whether they conceptualize the ego as facing toward the future or the past)?

(Experiment 3) Do temporal focus and age influence the choice between S‐Time and D‐Time among L1 Chinese speakers when interpreting temporally ambiguous expressions?

(Experiment 4) Do L1 English and L1 Chinese speakers differ in their reference‐frame preferences (S‐Time vs. D‐Time) for ambiguous temporal expressions, and are these differences primarily attribute to cross linguistic differences rather than demographic variables?

## 2 Experiment 1: Comparison of Temporal Focus Between Younger and Older L1 Chinese Speakers

### 2.1 Participants

All participants were native Chinese speakers. The younger adult group (aged 18–24, *n* = 50) comprised undergraduate and graduate students enrolled at the University of Macau, including 25 males and 25 females. Educational backgrounds in this group were highly homogeneous: 48 participants were current undergraduates and 2 were first‐year postgraduate students. The older adult group (aged 65–70, *n* = 50) consisted of healthy retirees recruited from Macau, including 23 males and 27 females. Their educational backgrounds were matched as closely as possible to those of the younger group, with 47 participants having completed their undergraduate degrees and 3 participants having completed secondary education (high school). All participants reported normal or corrected‐to‐normal vision and no history of neurological disorder.

The investigation secured formal ethical authorization through the Office of Research Services and Knowledge Transfer at the University of Macau (Ethics Assessment ID: SSHRE24‐APP047‐FAH). Operational protocols strictly aligned with internationally endorsed ethical benchmarks and jurisdiction‐specific regulatory statutes applicable to research with human participants. Adherence to these codified review mechanisms and protective measures ensured methodological compliance while prioritizing participant rights and welfare at all investigative stages.

### 2.2 Materials and Procedures

This study utilized the Temporal Focus Scale developed by Shipp et al. (2009) to examine the degree of focus on past and future time among younger and older Chinese adults under the influence of Chinese culture. Previous research by Liu and Zhang (2016) demonstrated that the Temporal Focus Scale is effective in measuring Chinese individuals' attention to different time periods (past vs. future). Similarly, Li and Cao (2018) successfully employed the Temporal Focus Scale to measure temporal focus across different ethnic groups in China.

The Temporal Focus Scale contains 12 items that assess past, present, and future focus. This study specifically focused on comparing past and future focus. Based on the methodologies of Liu and Zhang (2016) and Li and Cao (2018), eight Chinese‐translated items were selected, including four statements targeting past focus and four targeting future focus. Participants rated their responses to these items on a 7‐point Likert scale (1 = *not at all*, 7 = *extremely*), based on their immediate intuition.

To protect participants' privacy, they were provided with an informed consent form. Beginning the questionnaire was considered an indication of consent. Participants were also informed that they could withdraw from the study at any time during the response and data collection process.

### 2.3 Results

After collecting the questionnaires, the true purpose and background of the experiment were disclosed to participants. None of the participants correctly guessed the actual purpose of the study. Therefore, no data were excluded during processing and analysis.

**FIGURE 3:**
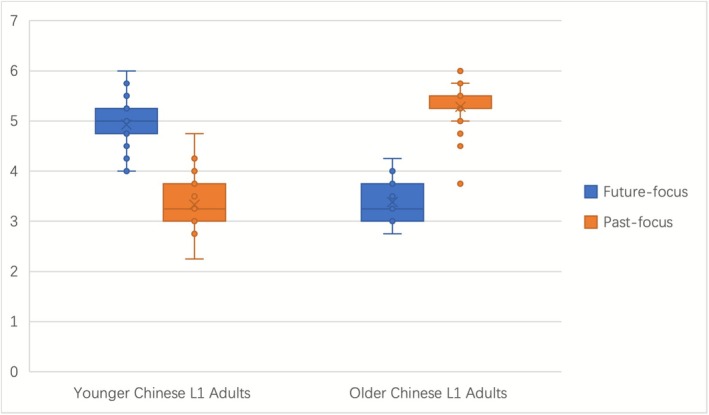
Comparison of temporal focus between younger and older L1 Chinese adults.

The data presented in Figure 3 indicate that younger adults scored higher on “future‐focused statements” compared to older adults, while older adults scored higher on “past‐focused statements” compared to younger adults. Specifically, the average score for younger adults on “future‐focused statements” exceeded 4, whereas their average score on “past‐focused statements” was below 4. Conversely, the average score for older adults on “past‐focused statements” exceeded 4, while their average score on “future‐focused statements” was below 4. Based on these findings, younger adults were categorized as the “future‐focused group”, and older adults were categorized as the “past‐focused group”.

Since the scores for younger and older adults on “past‐focused statements” did not meet the assumption of homogeneity of variances, Welch's t‐test was used for statistical analysis. The results showed Welch's t = −23.78, *p* < 0.001, and Cohen's d = 4.76, indicating a significant difference between younger and older adults in agreement with “past‐focused statements”, with older adults showing significantly greater past focus than younger adults.

For “future‐focused statements”, the scores for younger and older adults satisfied the assumption of homogeneity of variances. Therefore, an independent samples t‐test was conducted. The results showed t = 20.236, *p* < 0.001, and Cohen's d = 4.05, indicating a significant difference between younger and older adults in agreement with “future‐focused statements”, with younger adults showing significantly greater future focus than older adults.

To rule out potential confounding effects, an independent‐samples t‐test was conducted to examine whether temporal focus scores differed by gender. No significant differences were found for either future focus (t = 0.41, *p* = 0.68) or past focus (t = 0.29, *p* = 0.77). Education level was controlled at the recruitment stage, with the two age groups matched as closely as possible in their educational backgrounds, thereby eliminating the need for further statistical testing of this variable. These results indicate that gender and temporal focus were not significantly associated, supporting the validity of the subsequent group comparisons.

## 3 Experiment 2: Comparison of Ego‐Facing Direction Between Past‐Focused and Future‐Focused L1 Chinese Speakers

### 3.1 Participants

Based on the results of Experiment 1, we categorized 100 participants into two groups: the past‐focus group (participants with an average score > 4 on the four past‐focus statements and an average score < 4 on the four future‐focus statements) and the future‐focus group (participants with an average score > 4 on the future‐focus statements and an average score < 4 on the past‐focus statements). During the grouping process, we identified 5 younger and 3 older participants whose average scores on both the past‐focus and future‐focus dimensions were ≥ 4, and 1 older participant whose scores on both dimensions were ≤ 4. Given that these 9 participants had scores that were relatively balanced across both dimensions, making it difficult to clearly classify them into either group, we excluded their data from further analysis. After this adjustment, 91 participants from Experiment 1 proceeded to Experiment 2. Among them, 45 younger participants were classified into the future‐focus group, and 46 older participants were classified into the past‐focus group.

### 3.2 Materials and Procedures

This study employed a self‐designed questionnaire to assess the ego's tendency to face toward the future or past. The questionnaire consisted of 8 statements: 4 statements in which the future is in front of the ego and the past is behind the ego, and 4 statements in which the past is in front of the ego and the future is behind the ego (see Table 1). Participants were asked to rate each statement on a seven‐point Likert scale ranging from 1 (strongly disagree) to 7 (strongly agree). The ratings were used to determine whether the participants tended to face toward the past or the future. The study included two groups: one with a focus on the past and another with a focus on the future. This design aimed to measure whether there was a significant difference in the ego's facing between these two groups.

**TABLE 1:** Questionnaire items in experiment 2.

	Question number	Temporal statement
Future in front	Q1	I feel that I am facing the future
		我感觉我是面朝未来的。
	Q3	The past is behind me
		过去在我的身后。
	Q5	Yesterday is behind me
		昨天在我的身后。
	Q6	Tomorrow is in front of me
		明天在我的面前。
Past in front	Q4	I feel that I am facing the past
		我感觉我面朝着过去。
	Q8	The future is behind me
		未来來在我的身后。
	Q2	Tomorrow is behind me
		明天在我的身后。
	Q7	Yesterday is in front of me
		昨天在我的面前。

The internal consistency of the questionnaire was assessed using Cronbach's alpha, which yielded a value of 0.73. This indicates an acceptable level of internal consistency among the items. The validity of the questionnaire was evaluated through the Kaiser‐Meyer‐Olkin (KMO) measure of sampling adequacy, which produced a value of 0.76, indicating that the sample size was suitable for factor analysis. Bartlett's test of sphericity was significant (*p* < 0.001), suggesting that the data were appropriate for further analysis.

To protect participants' privacy, they were provided with an informed consent form. Beginning the questionnaire was considered an indication of consent. Participants were also informed that they could withdraw from the study at any time during the response and data collection process.

### 3.3 Results

After collecting the questionnaires, we disclosed the true purpose and background of the experiment to the participants. None of the participants correctly identified the actual purpose of the study. As a result, no data were excluded during processing and analysis.

**FIGURE 4:**
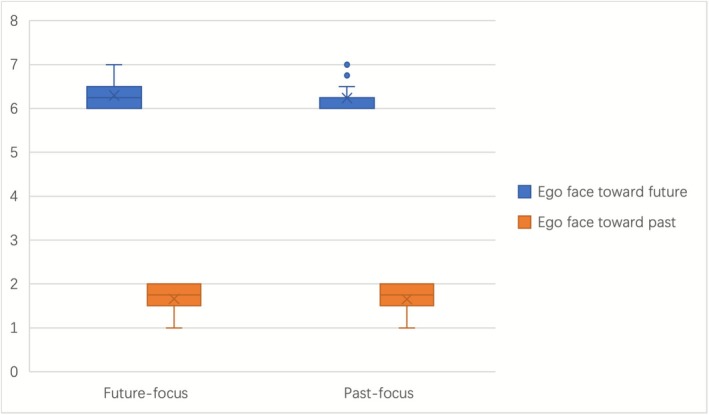
Comparison of ego between past‐focused and future‐focused L1 Chinese adults.

The results, as shown in Figure 4, indicate that both younger and older adults, regardless of their temporal focus, generally agreed with the statement “ego faces toward the future” and disagreed with “ego faces toward the past”. To further explore whether there were differences in ego orientation between native Chinese speakers with different temporal focuses when using ego as a reference to perceive time, we conducted t‐tests.

For scores on “ego faces toward the future”, the assumption of homogeneity of variances was satisfied, and an independent samples t‐test was performed. The results showed t = 0.718, *p* = 0.475 > 0.05, and Cohen's d = 0.15, indicating no significant difference between the two groups in agreement with “ego faces toward the future”. For scores on “ego faces toward the past”, the assumption of homogeneity of variances was not met, so Welch's t‐test was used. The results showed *p* = 0.280 > 0.05 and Cohen's d = 0.23, also indicating no significant difference between the two groups in agreement with “ego faces toward the past”.

In summary, both younger and older adults predominantly conceptualized time with the future in front of the ego and the past behind the ego, suggesting a future‐oriented temporal orientation.

## 4 Experiment 3: Comparison of Temporal Frame Selection Between Past‐Focused and Future‐Focused L1 Chinese Speakers

### 4.1 Participants

The participants in Experiment 3 were the same as in Experiment 2.

### 4.2 Materials and Procedures

In this study, we designed two tasks based on the interpretation of ambiguous sentences in Chinese to examine whether past‐focus and future‐focus participants differ in their temporal representations. Additionally, we aimed to analyze whether inconsistent temporal representations are due to differences in the ego's orientation (ego face toward the past or future). Specifically, the first task involved the phrase “前面的日子” (“qian mian de ri zi”, days in the front), asking participants to intuitively judge whether this phrase referred to the past or the future. The second task centered around the phrase “更好的日子在未来” (“geng hao de ri zi zai wei lai,” better days in the future), requiring participants to choose intuitively between “前面” (“qian mian”, front) or “后面” (“hou mian”, back) to represent future time. The Chinese items and choices employed in these tasks are listed in Table 2 (see the Chinese column); the table also provides the corresponding English translations.

**TABLE 2:** Questionnaire items and choices in experiment 3 and experiment 4.

Task	Description
Question 1	Days in the front 前面的日子
Option A	Past 过去
Option B	Future 未来
Question 2	Better days lie in the future. 更好的日子在未来
Option A	We will enjoy better days ahead. 更好的日子在前面
Option B	We will enjoy better days later. 更好的日子在后面

To protect participants' privacy, they were provided with an informed consent form. Beginning the questionnaire was considered an indication of consent. Participants were also informed that they could withdraw from the study at any time during the response and data collection process.

### 4.3 Results

After collecting the questionnaires, we disclosed the true purpose and background of the experiment to the participants. None of the participants correctly identified the actual purpose of the study. Consequently, no data were excluded during processing and analysis.

The data were initially examined to determine the distribution of responses across the two age groups. Specifically, we analyzed the frequency and percentage of responses in two categories: “ego reference” and “sequence reference”. As shown in Table 3, among younger (future‐focus) participants, 18 responses (40.00%) were classified as “前面的日子” (“qian mian de ri zi”, days in the front) representing the future, while 27 responses (60.00%) were categorized as past. In contrast, among older (past‐focus) participants, only 8 responses (17.39%) were identified as “前面的日子” (“qian mian de ri zi”, days in the front) for the future, with the remaining 38 responses (82.61%) attributed to past. Overall, across all participants, 26 responses (28.57%) fell under the future category, and 65 responses (71.43%) were assigned to past.

**TABLE 3:** Comparison of temporal direction between future‐focused and past‐focused L1 Chinese adults.

前面 (“qian mian”, front)	Future‐focused	Past‐focused	Sum
Future (ego reference)	18 (40.00%)	8 (17.39%)	26 (28.57%)
Past (sequence reference)	27 (60.00%)	38 (82.61%)	65 (71.43%)

Responses from L1 Chinese speakers with different temporal focuses to the first question, which asked whether “前面”(“qian mian”, front) represents the past or the future, were analyzed using a chi‐square test. The results showed Pearson χ^2^ = 5.70, *p* < 0.05, indicating a significant difference between the past‐focused and future‐focused groups in their interpretation of the temporal directionality of “前面”(“qian mian”, front) (whether it represents the past or the future). Specifically, younger participants with a future focus were more likely to interpret “the days in front” as referring to the future compared to older participants with a past focus. This statistically significant difference suggests that both age and temporal focus are associated with the representation of temporal directionality.

However, despite the significant cognitive differences, a common pattern was observed: both past‐focused and future‐focused Chinese speakers generally associated “the days in front” with the past.

Based on the conclusions of Experiment 2, we know that ego towards the future is the cognitive preference for both past‐focused and future‐focused Chinese speakers. In Experiment 3, participants from both groups tended to associate “前面” (“qian mian”, front) with the past, which seems to contradict the conclusion of Experiment 2 that “past is behind the ego”. This apparent contradiction can be explained by the fact that in this context, “前面” (“qian mian”, front) represents earlier in time, which corresponds to the past. This conceptualization does not involve the ego as a reference point, meaning there is no question of ego towards the past or ego towards the future. Instead, this is S‐Time, where earlier events are perceived as being in front.

Among L1 Chinese speakers, the observer's perspective remains ego‐focused towards the future, but past‐focused participants are more likely than future‐focused participants to associate “the days in front” with the past. This tendency is influenced by multiple factors, reflecting individual cognitive preferences in the choice of reference frames among Chinese speakers.

Overall, whether participants were past‐focused older adults or future‐focused younger adults, Chinese speakers tended to use S‐Time, interpreting “the days in front” as “earlier days”, that is, days in the past. Therefore, Chinese speakers predominantly employ S‐Time, with ego perspectives being less popular in their cognitive processing.

The data were initially examined to understand how participants represent the future. Table 4 displays the distribution of responses between younger and older Chinese adult participants regarding whether “前面” (“qian mian”, front) or “后面” (“hou mian”, back) represents future days. Among younger participants, 22 individuals (48.89%) chose “前面” (“qian mian”, front) to indicate future days, while 23 individuals (51.11%) opted for “后面” (“hou mian”, back). In contrast, among older participants, only 11 individuals (23.91%) selected “前面” (“qian mian”, front) for the future, whereas a larger proportion, 35 individuals (76.09%), preferred “后面” (“hou mian”, back). Overall, after combining both age groups, 33 responses (36.26%) were in the “前面” (“qian mian”, front) category and 58 responses (63.74%) were in the “后面” (“hou mian”, back) category. This suggests that L1 Chinese speakers prefer to use “后面” (“hou mian”, back) to represent the future and tend to rely on sequence reference to characterize temporal direction.

**TABLE 4:** Representation of future between future‐focused and past‐focused L1 Chinese adults.

未来 (“wei lai”, future)	Younger	Older	Sum
前面(“qian main”, front)	22 (48.89%)	11 (23.91%)	33 (36.26%)
后面 (“hou mian”, back)	23 (51.11%)	35 (76.09%)	58 (63.74%)

For the second question, which asked whether “前面” (“qian mian”, front) or “后面” (“hou mian”, back) represents the future, data from past‐focused and future‐focused Chinese participants were analyzed using a chi‐square test. The results showed Pearson χ^2^ = 6.14, *p* < 0.05, indicating a significant difference in how the two groups perceive temporal directionality for the future. Specifically, younger participants with a future focus were more likely to use “front” to represent future days compared to older participants with a past focus. This statistically significant difference highlights that temporal representation among Chinese speakers is not uniform. It varies with participants' age and their dominant temporal focus.

Despite these differences, a common pattern emerged: both past‐focused and future‐focused Chinese speakers showed a preference for using “后面” (“hou mian”, back) to represent future days.

Similar to the first question, participants who used “后面” (“hou mian”, back) to represent the past adopted D‐Time, where the past is perceived as being behind the ego (the past is behind the ego). In contrast, participants who used “前面” (“qian mian”, front) to represent the future relied on S‐Time, where “后面” (“hou mian”, back) signifies a relatively later point in time, which corresponds to the future in this context.

Among L1 Chinese speakers, the observer's perspective remains consistently ego‐focused towards the future, and past‐focused participants were more likely than future‐focused participants to use “后面” (“hou mian”, back) to represent “future days”. Overall, regardless of whether participants were past‐focused older adults or future‐focused younger adults, Chinese speakers tended to employ S‐Time and preferred to use “后面” (“hou mian”, back) to represent “future days”. This further supports the idea that Chinese speakers predominantly adopt S‐Time.

Our results indicate that temporal focus alone does not fully determine speakers' linguistic temporal representations. Crucially, however, we distinguish two distinct phenomena that should not be conflated. The first is ego directionality, which refers to whether the ego is conceptualized as facing toward the future or the past. The second is reference‐frame preference, which concerns how ambiguous directions are interpreted—specifically, whether via S‐Time or D‐Time. In this study, ego directionality was uniform across Chinese age groups (see Experiment 2), with both younger and older participants predominantly adopting a future‐facing ego perspective. By contrast, age and temporal focus affected reference‐frame preference (see Experiment 3), with older participants showing a stronger tendency toward S‐Time. We therefore treat age differences as differences in reference‐frame selection, not as differences in basic ego directionality.

## 5 Experiment 4: Comparison of Temporal Frame Selection Between L1 Chinese and L1 English Speakers

### 5.1 Participants

Based on research by Clark (1973), McGlone and Harding (1998) and Boroditsky (2000), it has been established that native English speakers typically have a future temporal focus, with a self‐orientation towards the future (ego moving or time moving). This experiment aims to investigate the temporal direction representation preferences of future‐oriented L1 English speakers. Additionally, the results of this experiment will be compared with the temporal representation data of L1 Chinese speakers from Experiment 3, to explore whether differences in linguistic backgrounds significantly influence participants' temporal representations and whether there are notable differences in temporal representation preferences between Chinese and English speakers.

For this experiment, 90 native English speakers, aged 18–65, were randomly recruited from public locations in Macao. To ensure demographic comparability with the L1 Chinese speakers, participants were classified into age groups paralleling the Chinese sample: younger adults (aged 18–30 years, *n* = 48) and older adults (aged 65–70, *n* = 42). The L1 English participants comprised 45 males and 45 females, with balanced gender distributions in each age group (younger: 22 males, 26 females; older: 20 males, 22 females). Educational backgrounds were matched to those of L1 Chinese speakers: all younger participants were current undergraduates, whereas older participants held undergraduate (*n* = 40) or had completed secondary education (high school) (*n* = 2). All participants reported normal or corrected‐to‐normal vision and no history of neurological disorders.

### 5.2 Materials and Procedures

The experimental materials consisted of an English version of the survey used in Experiment 3, designed to examine native English speakers' preferences for temporal representations and reference frame selection.

The experiment included two tasks: In the first task, participants were asked to intuitively judge whether “days in the front” referred to the past or the future. In the second task, participants read the statement “Better days lie in the future” and selected one of two sentences that they felt best conveyed this meaning. The options provided were: “We will enjoy better days ahead.” and “We will enjoy better days later.” Full questionnaire details (Chinese and English items and response options) are provided in Table 2.

To protect participants' privacy, all participants were asked to read an informed consent form. Starting the task was considered consent to participate. Participants were informed that they could withdraw from the experiment at any time during the response or data collection process.

### 5.3 Results

After collecting the questionnaires, we debriefed participants about the true purpose and background of the experiment. None of the participants guessed the actual purpose of the study. Therefore, no data were excluded during processing and analysis.

Preliminary analyses in Table 5 showed that 28.57% of L1 Chinese speakers interpreted “前面” (“qian mian”, front) as referring to the future, while 71.43% interpreted it as referring to the past (S‐Time). In contrast, 98.89% of L1 English speakers took “front” to mean the future (D‐Time), with only 1.11% interpreting it as the past. Overall, Chinese speakers predominantly employ S‐Time—conceptualizing “前” (“qian”, front) as denoting the past—whereas English speakers predominantly employ D‐Time—conceptualizing “front” as denoting the future.

**TABLE 5:** Distribution of representation of “前” (“qian”, front) between L1 Chinese and L1 English adults.

前面(“qian main”), front	Future	Past
Chinese	26 (28.57%)	65 (71.43%)
English	87 (98.89%)	3 (1.11%)

The responses of L1 Chinese and L1 English speakers to whether “前面” (“qian mian”, front) represents the past or the future were analyzed using a chi‐square test. The results showed Pearson χ^2^ = 89.46, *p* < 0.01, indicating a significant difference in temporal direction cognition between Chinese and English speakers. Chinese speakers tended to interpret “前面的日子” (“qian mian de ri zi”, days in the front) as referring to the past, whereas English speakers interpreted it as referring to the future. These findings suggest that, while age and temporal focus have some influence on temporal linguistic cognition in Chinese speakers, the primary factor underlying the differences between responses by L1 Chinese and L1 English speakers is the linguistic divergence between Chinese and English. The differences in dominant cognitive patterns between the two languages play a crucial role in their respective temporal direction representations.

As shown in Table 6, preliminary analyses showed that 28.57% of L1 Chinese speakers interpreted “前面” (“qian mian”, front) as referring to the future, while 71.43% interpreted it as referring to the past (S‐Time). In contrast, 98.89% of L1 English speakers took “front” to mean the future (D‐Time), with only 1.11% interpreting it as the past. Overall, Chinese speakers exhibited a clear preference for S‐Time—using “后面” (“hou mian”, back, means later) to denote the future—whereas English speakers predominantly employed D‐Time—using “front” to denote the future.

**TABLE 6:** Distribution of representation of future between L1 Chinese and L1 English adults.

Future	Chinese	English
前面 (front)	33 (36.26%)	76 (84.44%)
后面 (back)	58 (63.74%)	14 (15.56%)

Similarly, the responses of Chinese and English speakers to whether they use “前面” (“qian mian”, front) or “后面” (“hou mian”, back) to represent the future were analyzed using a chi‐square test. The results showed Pearson χ^2^ = 43.85, *p* < 0.01, indicating a significant difference in temporal direction representation between the two groups. Chinese speakers were more likely to associate the future with “后面” (“hou mian”, back), whereas English speakers associated the future with “front”.

In conclusion, while age and temporal focus have some influence on temporal direction representation, the differences in dominant cognitive patterns between L1 Chinese and L1 English are the primary drivers of the observed variations in temporal direction representation.

## 6 Discussion

The findings from our experiments reveal how age, cultural temporal focus, and linguistic frameworks shape time cognition in L1 Chinese speakers. While age influences temporal priorities—younger adults emphasize the future and older adults the past—both groups share a future‐oriented self‐perspective. This challenges the presumption that conflates Chinese temporal representation and cognition—such as the use of “前” (“qian”, front) to denote the past—with a culturally ingrained past‐facing temporal orientation (Alverson 1994; Li and Zhang 2017).

The data from Experiment 1 revealed significant differences in the emphasis on the past and future between younger and older native Chinese speakers. Younger Chinese speakers showed a stronger focus on the future, while older Chinese speakers prioritized the past. Further analysis based on temporal focus scores allowed us to categorize participants into future‐focused and past‐focused groups, with only 9 datasets remaining unclassified. Notably, the future‐focused group consisted entirely of younger adults (*n* = 45), and the past‐focused group comprised only older adults (*n* = 46). This finding refines previous generalizations about Chinese people predominantly valuing the past (Alverson 1994; Li and Zhang 2017). Through subgroup analysis, we demonstrated that older native Chinese speakers indeed focus on the past, while younger Chinese adults are significantly more future‐oriented. Thus, whether Chinese people emphasize the past or future cannot be oversimplified, as age is a critical influencing factor.

Prior research attributes Chinese speakers' preference for using “前” (“qian”, front) to denote the past to cultural values centered on historical reverence, suggesting that this linguistic feature implies a past‐facing temporal orientation (Alverson 1994; Li and Zhang 2017). Experiment 1 confirmed that older Chinese speakers emphasize the past, but does this mean they face toward the back? Do younger future‐focused and older past‐focused individuals face toward opposite temporal directions? Experiment 2 addressed this by having native Chinese speakers rate statements positioning the ego as facing the future (back to the past) or facing the past (back to the future). Results showed no significant difference in temporal orientation between groups: both past‐ and future‐focused Chinese speakers perceived themselves as facing the future, aligning with English speakers' ego orientation (Yu 1998; Chen 2021a; Bao 2022; Yang et al. 2023). When contextualized with an explicit self‐reference, both groups consistently oriented toward the future, with the past behind them.

Experiment 2 demonstrated that both past‐ and future‐focused Chinese speakers face the future. Experiment 3 further revealed that participants across temporal focus groups predominantly interpreted “前面的日子” (“qian mian de ri zi”, days in front) as referring to the past and used “后面” (“hou mian”, back) to represent the future. Thus, from a D‐Time perspective, “前面” (“qian mian”, front) corresponds to the observer's forward‐facing direction (future), while “后面” (“hou mian”, back) denotes the past. The use of “前面” (“qian mian”, front) for the past and “后面” (“hou mian”, back) for the future arises from S‐Time, where earlier times are “front” and later times “back” (Yu 2012; Zhang 2003; Núñez et al. 2006; Chen 2021a; Chen 2021b). This future‐facing temporal cognition among Chinese speakers does not conflict with “前” (“qian”, front) denoting the past, as S‐Time dominates over D‐Time in Chinese (Yu 2012; Chen 2021a; Yang et al. 2023). The ambiguous sentences in Experiment 3 failed to activate D‐Time in most participants, avoiding issues of ego orientation.

Despite significant differences in temporal representation between past‐ and future‐focused Chinese speakers, S‐Time emerged as their shared dominant cognitive mode. The asymmetry between “前” (“qian”, front) and “后” (“hou”, back) in modern Chinese—where “前” (“qian”, front) can denote both past and future, while “后” (“hou”, back) exclusively marks the future (Shen 1999)—further underscores the primacy of S‐Time. This linguistic asymmetry reinforces the conclusion that S‐Time is the default and dominant cognitive framework in Chinese.

Thus, when D‐Time contexts are explicitly activated, L1 Chinese speakers' temporal cognition remains toward the future, unaffected by temporal focus differences. Both L1 Chinese and L1 English speakers share a consistent ego‐facing direction (Yu 1998; Chen 2021a; Bao 2022). However, Experiments 3 and 4 revealed stark contrasts in dominant temporal reference frames between the two languages. In ambiguous contexts, Chinese speakers prioritize S‐Time, associating “前” (“qian”, front) with the past, while English speakers default to D‐Time, linking “front” with the future. Chinese speakers' use of “前” (“qian”, front) for the past does not indicate a past‐facing orientation but reflects the dominance of S‐Time in unmarked contexts (lacking explicit self‐perspective cues).

This study finds that, in terms of temporal‐direction cognition, the cognitive style associated with Chinese is S‐Time, whereas that associated with English is D‐Time. Temporal direction expressions are closely related to these cognitive styles. This raises a further question: since both S‐Time and D‐Time are constructed upon a universal psychological foundation, why do Chinese and English display significant differences in their cognitive styles?

Cognition, as a mediating variable between language and culture (Levinson 1996), establishes a close connection among the three. Language is comprehended and employed through cognitive processes, while at the same time reflecting distinct cognitive patterns. Meanwhile, cultural backgrounds and their underlying philosophical values profoundly influence and even shape the cognitive styles of a cultural group or nation (Chen 2021a). A nation's cognitive style regarding temporal direction is inevitably influenced by its philosophical values.

The relationship between subject and object has long been one of the core issues in philosophy, and it plays a crucial role in shaping the cognitive styles of a people and their language. This relationship profoundly influences how different cultures conceptualize time. In Chinese culture, the philosophical outlook and values of “the unity of heaven and humanity” emphasize the harmonious integration of subject and object, viewing human beings as part of the objective world. Accordingly, in the cognition and expression of time, Chinese tends to prioritize the object (i.e., the inherent properties of time and its natural sequence) as the reference point, thereby forming the S‐Time. In contrast, the Western philosophical tradition highlights the “subject–object dichotomy”, advocating that “man is the measure of all things,” which underscores the centrality of the subject and frames the understanding and representation of objects from the perspective of the self. This philosophical orientation leads English to conceptualize time primarily with the subject (i.e., ego) as the reference point, thereby forming the D‐Time.

In summary, this study empirically demonstrates that age influences temporal focus among Chinese speakers, with older individuals emphasizing the past and younger ones the future. Crucially, even past‐focused older Chinese speakers maintain a future‐oriented temporal cognition. This challenges the notion that Chinese cultural reverence for history implies a past‐facing temporal orientation (Li and Zhang 2017; Gu et al. 2019). Instead, the preference for “前” (“qian,”, front) to denote the past aligns with the primacy of S‐Time in Chinese, contrasting with English's D‐Time. These distinct dominant reference frames in temporal representation between Chinese and English stem from deeper philosophical foundations: Chinese culture's emphasis on “unity of heaven and humanity” fosters S‐Time, while Western “subject‐object dichotomy” prioritizes D‐Time.

## 7 Conclusion

This study yields several key insights into how temporal focus, ego orientation, and reference‐frame selection interact across languages and age groups:

Age significantly modulates temporal focus among Chinese speakers: younger adults exhibit a stronger future focus, whereas older adults prioritize the past. Despite these differences in temporal focus, both past‐ and future‐focused Chinese groups consistently maintain a future‐facing ego orientation when using D‐Time. The seemingly paradoxical use of “前” (“qian”, front) to denote the past in Chinese stems from the dominance of S‐Time—earlier events are conceptualized as “front” and later events as “back”, independent of the ego's orientation. In contrast, English speakers overwhelmingly employ D‐Time, consistently mapping “front” onto the future.

The culture and philosophical outlook of a people shape their cognitive style, and the differences in philosophical values between Chinese and English speakers have given rise to distinct cognitive styles in the two languages. However, although this study highlights the important role of philosophical ideas and cultural values in shaping temporal cognitive styles, the specific mechanisms and scope of their influence remain to be further explored. It would be valuable for future research to examine how different philosophical traditions and cultural values are internalized as shared cognitive tendencies—through processes of socialization, linguistic practices, and the transmission of cognitive strategies—and how they thereby form stable cognitive styles. Moreover, whether the influence of philosophy and culture on cognition is likewise reflected in other domains (e.g., space, event structure, word order), that is, whether such influence is limited to particular domains or exhibits a broader cognitive universality, constitutes a crucial direction for future investigation.

## Disclosure

The author has nothing to report.

## Ethics Statement

This study was conducted in accordance with the ethical standards and was approved by the Office of Research Services and Knowledge Transfer at the University of Macau (Ethics Assessment ID: SSHRE24‐APP047‐FAH). Written informed consent was obtained from all participants.

## Conflicts of Interest

The author declares no conflicts of interest.

## Data Availability

The anonymized raw data generated in this study may be shared for legitimate academic purposes under the condition of strict adherence to ethical review requirements and participant privacy protection protocols. Researchers interested in accessing the dataset may submit a data request via email to the corresponding author, Rong Bao (email: yc27730@um.edu.mo). The provided data have undergone rigorous de‐identification processes, wherein all direct or indirect personal identifiers—including but not limited to names, contact information, and geographic locations—have been permanently removed to ensure irreversible anonymization.
